# PACAP/PAC1 regulation in cystitis rats: induction of bladder inflammation cascade leading to bladder dysfunction

**DOI:** 10.3389/fimmu.2024.1413078

**Published:** 2024-11-28

**Authors:** Hanwei Ke, Lin Zhu, Weiyu Zhang, Huanrui Wang, Zehua Ding, Dongyu Su, Qi Wang, Kexin Xu

**Affiliations:** ^1^ Department of Urology, Peking University People’s Hospital, Beijing, China; ^2^ Peking University Applied Lithotripsy Institute, Peking University People’s Hospital, Beijing, China; ^3^ Department of Plastic Surgery, Affiliated Beijing Chaoyang Hospital of Capital Medical University, Beijing, China

**Keywords:** interstitial cystitis, bladder pain syndrome, PACAP, PAC1 receptor, bladder inflammation

## Abstract

**Introduction:**

Interstitial Cystitis/Bladder Pain Syndrome (IC/BPS) is a chronic and debilitating condition marked by bladder pain, urinary urgency, and frequency. The pathophysiology of IC/BPS remains poorly understood, with limited therapeutic options available. The role of Pituitary Adenylate Cyclase-Activating Polypeptide (PACAP) and its receptor PAC1 in IC/BPS has not been thoroughly investigated, despite their potential involvement in inflammation and sensory dysfunction. This study aims to examine the expression and functional role of the PACAP/PAC1 signaling pathway in the pathogenesis of IC/BPS.

**Methods:**

Bladder tissue samples from IC/BPS patients and a rat model of cystitis were analyzed to evaluate PACAP and PAC1 expression. Transcriptomic analysis, immunohistochemistry, and bladder function assays were employed to assess the correlation between PACAP/PAC1 activation, bladder inflammation, and sensory dysfunction. Additionally, modulation of the PACAP/PAC1 pathway was tested in rats to determine its effects on bladder inflammation and function.

**Results:**

Our results demonstrate significant upregulation of PACAP and PAC1 in both human bladder tissues from IC/BPS patients and in the rat cystitis model. This upregulation was associated with increased bladder inflammation and sensory dysfunction. Intervention with PACAP/PAC1 pathway modulation in rats resulted in a marked reduction in bladder inflammation and improvement in bladder function, suggesting the pathway’s pivotal role in disease progression.

**Discussion:**

The findings provide compelling evidence that the PACAP/PAC1 pathway is involved in the inflammatory and sensory changes observed in IC/BPS. By targeting this signaling pathway, we may offer a novel therapeutic approach to mitigate the symptoms of IC/BPS. This study enhances our understanding of the molecular mechanisms driving IC/BPS and opens avenues for the development of targeted treatments.

## Introduction

Interstitial cystitis/bladder pain syndrome (IC/BPS) is a complex chronic inflammatory bladder disorder, marked by bladder pain, nocturia, urgency, sterile urine, and frequent urination (1, 2). Despite various hypotheses, the origin of IC/BPS remains unclear, and its pathophysiology is poorly understood (3). IC/BPS primarily affects females, with an estimated prevalence of 6.53% among women in the U.S (4). No therapeutic approach has yet consistently succeeded in providing lasting relief from IC/BPS symptoms (5). The exact origin and pathophysiology of IC/BPS remain unclear, though emerging research highlights bladder urothelial injury or dysfunction and a sustained inflammatory cycle as key factors (6, 7).

Pituitary adenylate cyclase-activating polypeptide (PACAP), a neuropeptide, plays a role in regulating lower urinary tract (LUT) functions (8–10). Part of the vasoactive intestinal polypeptide (VIP), secretin, and glucagon hormone family, PACAP shares about 68% homology with VIP. Neuropeptides such as PACAP are expressed in both neural and non-neural tissues of the LUT, including afferent neurons, neural pathways, plasma, inflammation or injury sites, bladder fibroblasts, detrusor muscle, and urothelium (11). PACAP immunoreactivity appears in the C-fiber bladder afferents of the dorsal root ganglia (DRG), bladder smooth muscle, sub-urothelial nerve plexus, and peri-vascular nerve fibers (12). Urothelial cells express the PACAP receptor PAC1, which releases ATP upon PACAP stimulation, activating receptors on sub-urothelial sensory nerve fibers (13). Braas et al. demonstrated PACAP’s role in micturition, emphasizing how inflammation-induced changes in peripheral and central micturition pathways can contribute to bladder dysfunction (10).

The pathophysiology of IC/BPS involves a complex sequence of inflammatory responses, possibly initiated or aggravated by neuropeptide dysregulation, including PACAP. However, the exact role and regulatory mechanisms of these neuropeptides in bladder inflammation and post-inflammatory dysfunction remain poorly understood. Although previous research has highlighted PACAP’s general involvement in inflammation and its potential relevance to IC/BPS, a substantial gap remains in understanding the specific mechanisms by which PACAP/PAC1 interactions impact the inflammatory cascade in IC/BPS. This study aims to narrow this gap by examining the regulatory role of PACAP/PAC1 in a rat model of bladder cystitis, focusing on PACAP induction, inflammation progression, and its effects on bladder function in IC/BPS.

## Materials and methods

### Ethical approval and informed consent

The research protocol was approved by the Ethics Committee of Peking University People’s Hospital and complies with the principles outlined in the Helsinki Declaration. The ethical approval number is 2022PHB400-001. All patients have provided informed consent.

### Sample collection

Bladder tissue specimens were collected from patients diagnosed with IC/BPS during cystoscopy and biopsy at Peking University People’s Hospital. The diagnostic criteria for IC/BPS were based on the guidelines of the American Urological Association (14). Control bladder specimens were taken from normal tissue adjacent to cancerous areas in patients with bladder cancer. Six Hunner-type interstitial cystitis (HIC) samples and three normal samples were used for transcriptome sequencing and immunohistochemical validation.

### Transcriptomic research methods of bladder biopsy

#### RNA extraction and sequencing

Tissue samples were collected, and RNA was extracted using the Trizol reagent kit (Invitrogen, Carlsbad, CA, USA) following the manufacturer’s guidelines. RNA quality was assessed with the Agilent 2100 Bioanalyzer (Agilent Technologies, Palo Alto, CA, USA). After total RNA extraction, eukaryotic mRNA was enriched using Oligo(dT). The mRNA was then fragmented and reverse-transcribed into cDNA using the NEBNext Ultra RNA Library Prep Kit for Illumina (NEB#7530, New England Biolabs, Ipswich, MA, USA), and sequenced on an Illumina Novaseq6000 platform by Gene Denovo Biotechnology Co. (Guangzhou, China).

#### Partial least squares-discriminant analysis

To investigate molecular differences between Hunner-type interstitial cystitis (HIC) patients and normal controls, we performed PLS-DA. This method identifies variables with high separation capability, focusing on differences in gene expression profiles from high-throughput RNA sequencing data. Before analysis, data were log-transformed and auto-scaled to stabilize variance and ensure comparability across samples.

#### Immunoinfiltration analysis

The extent and patterns of immune cell infiltration in bladder tissues were analyzed through immunohistochemistry. Specific markers for immune cells, such as CD4, CD8, and FoxP3, were used to stain tissue sections. High-resolution microscope images were captured, and the percentage of positively stained cells was quantified with ImageJ software. This approach provided insights into the immune landscape of HIC and normal bladder tissues.

#### RNA data analysis

Ribosomal RNA alignment was followed by genome alignment using HISAT2 and quantification with StringTie. FPKM values were calculated with RSEM software. Differential expression analysis between groups was conducted using DESeq2, applying criteria of fold change ≥ 2 and FDR < 0.05. Gene Ontology (GO) and Kyoto Encyclopedia of Genes and Genomes (KEGG) enrichment analyses of differentially expressed mRNAs (DEmRNAs) were performed using hypergeometric tests. Additionally, gene set enrichment analysis (GSEA) was used to determine the functions of differentially expressed genes between groups.

### Animals

Sprague-Dawley female rats (7 weeks old) were obtained from Janvier Labs. The animals were kept at a controlled temperature (21 ± 3°C) on a 12-hour light/dark cycle with free access to food and water. Rats were acclimated to laboratory conditions for at least 3 days before the start of experiments. At the end of the procedures, rats were euthanized humanely using CO2 inhalation (100%, 3 L/min), followed by cervical dislocation. All animal experiments were approved by the Medical Ethics Committee of Peking University People’s Hospital (Approval number: 2019PHE060).

### Induction of cystitis and drug treatments

**Figure 1:**
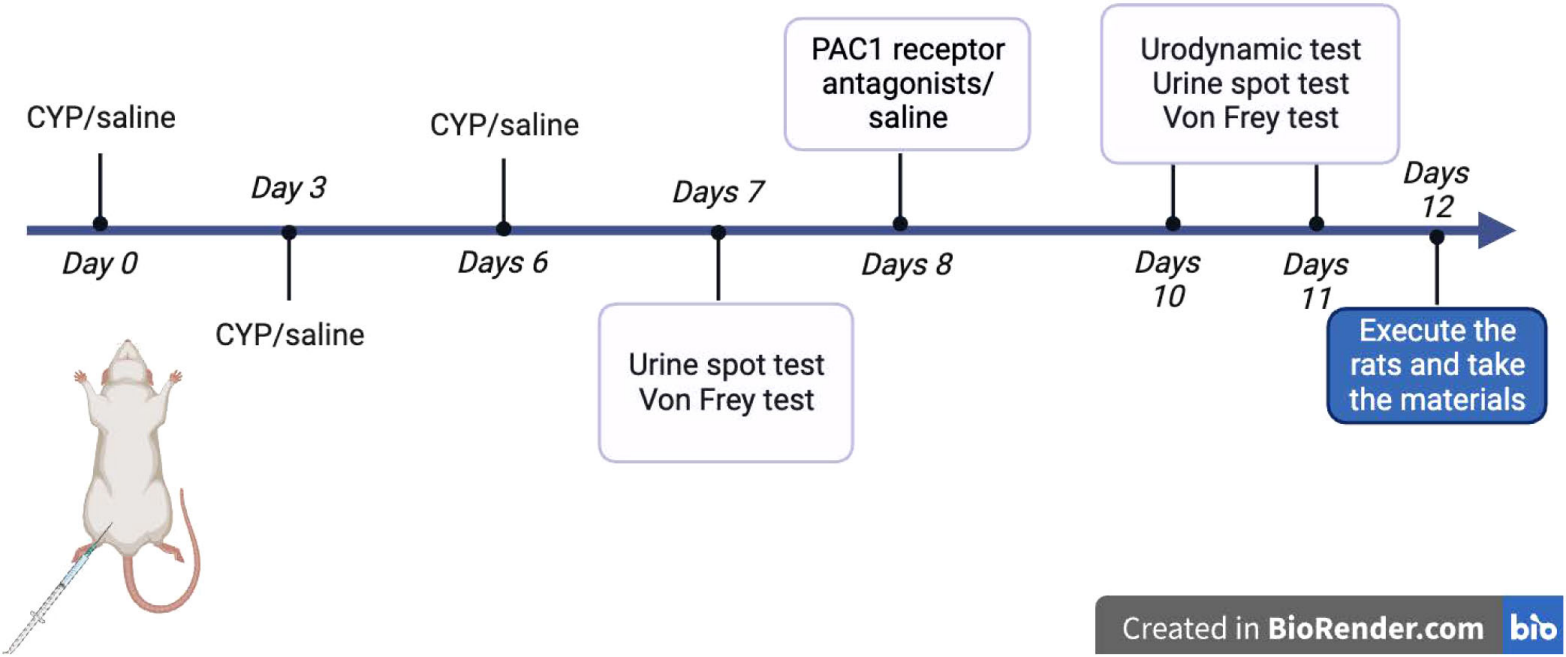
Schematic diagram of methodology for animal experiments: Schematic diagram illustrating the methodology for animal experiments conducted with Sprague-Dawley rats. This includes the steps of inducing chronic cystitis via intraperitoneal injections of cyclophosphamide (CYP), control treatments, and subsequent administrations of PACAP6-38, both intravesically and intrathecally, with specific concentrations. Tail flicking in response to intrathecal injections in the S1 spinal segment confirmed the accuracy of the procedure.

Chronic cystitis was induced in Sprague-Dawley rats through intraperitoneal injections of cyclophosphamide (CYP) from Sigma-Aldrich, St. Louis, MO, USA, at 25 mg/kg every third day (days 0, 3, 6). Control rats received intraperitoneal injections of 0.9% NaCl saline (5 ml/kg) under the same conditions. The study included 42 rats, randomized into six groups: Con-Con (intraperitoneal and intravesical saline), CYP-Con (intraperitoneal CYP and intravesical saline), CYP-PAC (intraperitoneal CYP and intravesical PACAP6-38), Con-PAC (intraperitoneal saline and intravesical PACAP6-38), CYP-Treated (intraperitoneal CYP and intrathecal PACAP6-38), and CYP-Untreated (intraperitoneal CYP and intrathecal saline). PACAP6-38, a potent PAC1 receptor antagonist (15), was administered at 300 nM for intravesical and 50 nM for intrathecal administration to explore its therapeutic potential in alleviating symptoms associated with interstitial cystitis, particularly those induced by cyclophosphamide (CYP). Intravesical infusion was performed under anesthesia with a clamped urethra for 30 minutes. Intrathecal injection was conducted at the S1 spinal segment, with tail flicking indicating successful puncture. These procedures were carried out two days post-modeling. Intravesical infusion was performed under anesthesia with a clamped urethra for 30 minutes. Intrathecal injection was conducted at the S1 spinal segment, with tail flicking indicating successful puncture. These procedures were carried out two days post-modeling (**Figure 1**).

### Von Frey filaments test

Following the third CYP or saline bladder instillation and subsequent drug treatment, nociceptive responses were assessed. Mechanical stimulation of the lower abdomen near the bladder was conducted using eight von Frey filaments with progressively increasing force (North Coast, USA), following previous protocols (16). To maintain consistency in pain testing, all tests were conducted by a single experimenter under standardized conditions. Before testing, the designated abdominal area on each rat was shaved. The rats were placed in individual transparent Plexiglas boxes on an elevated wire mesh floor and acclimated for at least 30 minutes. During the von Frey test, each filament was applied through the mesh for 1–2 seconds with sufficient force to bend slightly. This process was repeated three times for each filament with a 5-second interval between applications, and care was taken to vary the stimulated areas near the bladder to prevent desensitization. Nociceptive response scoring was defined as follows: 0 = no response; 1 = abdominal retraction; 2 = retraction plus position change; 3 = retraction, position change, licking the stimulated area, and/or vocalization. The nociceptive score was calculated as a percentage of the maximum possible score from the three pooled applications (17).

### Urination patterns

During the experiment, Sprague-Dawley (SD) rats were individually placed in standard cages for 1 hour, with the bedding replaced by Whatman Grade 3 filter paper. The rats had unrestricted access to food and water. Urine spots were photographed under UV light to measure the percentage of the area they covered. The area of the urine spots was analyzed and calculated using ImageJ software.

### Filling cystometry

Before the experiment, air is removed from the infusion pump and tubing, and the pump is set to 6 mL/h. Anesthesia is induced in the rat using inhaled isoflurane and maintained with an animal face mask while the rat is placed in a supine position on the platform. The urethral orifice is disinfected, and a 19G pressure catheter is carefully inserted 3 cm deep into the bladder through the urethra and secured with adhesive tape. With a physiological saline infusion, urinary dynamics are monitored via computer software, and data is recorded using the pressure gauge. The experiment begins once the urinary dynamic curve stabilizes.

### Immunohistochemistry

Paraffin sections were deparaffinized and rehydrated using dewaxing solution and graded ethanol. Antigen retrieval was subsequently performed through microwave treatment in citrate buffer (pH 6.0). After natural cooling, sections were rinsed with PBS and blocked for endogenous peroxidase using a 3% hydrogen peroxide solution. Serum blocking with 3% BSA was conducted, and primary antibodies were applied and incubated overnight at 4°C. After washing, appropriate secondary antibodies were applied, and the sections were incubated at room temperature. DAB staining followed, monitored under a microscope, and was stopped with tap water rinsing. Counterstaining with hematoxylin, graded dehydration, and mounting with coverslips were followed by microscopic examination for results. Aipathwell software was used for automated positioning, positive expression determination, and H-SCORE calculation based on staining intensity percentages. (H-SCORE = ∑(pi × i) = (percentage of weak intensity × 1) + (percentage of moderate intensity × 2) + (percentage of strong intensity × 3)).

### Bladder inflammation assessment and histopathology

Animals were sacrificed at the indicated times after the first injection of CYP or saline. Urinary bladders were quickly collected and assessed for bladder weight, wall thickness, and edema evaluation. Urinary bladders were quickly collected and assessed for bladder weight, wall thickness, and edema evaluation. Each bladder was examined macroscopically for edema and scored based on criteria established by Gray et al. (18) as follows: absent (0), mild (1), moderate (2), and severe (3). Edema was classified as severe when fluid was observed both externally and internally on the bladder wall. Edema confined to the internal mucosa was classified as moderate. Edema between normal and moderate was defined as mild. Bladders were fixed in 10% formalin and embedded in paraffin. Bladder sections were stained with hematoxylin and eosin (HE) and digitized using a slide scanner (Nanozoomer, Hamamatsu, objective ×20).

### Statistical analysis

Statistical analysis and visualization in this study were conducted using R software (version 3.6.3). For normally distributed quantitative data, results were presented as mean ± standard deviation, while non-normally distributed data were represented as median and interquartile range. Comparisons between two groups with normally distributed data were assessed using an independent samples t-test, while a paired t-test was used for pre- and post-treatment efficacy comparisons. For data that did not pass the Shapiro-Wilk normality test, statistical analysis was performed using the Wilcoxon rank sum test. For data that did not meet the normality assumptions for multiple groups, the Kruskal-Wallis rank sum test was used, and significance was corrected with the Bonferroni method.

## Result

### Elucidating the immunopathogenesis of IC/BPS: insights from molecular profiling and pathway analysis

**Figure 2:**
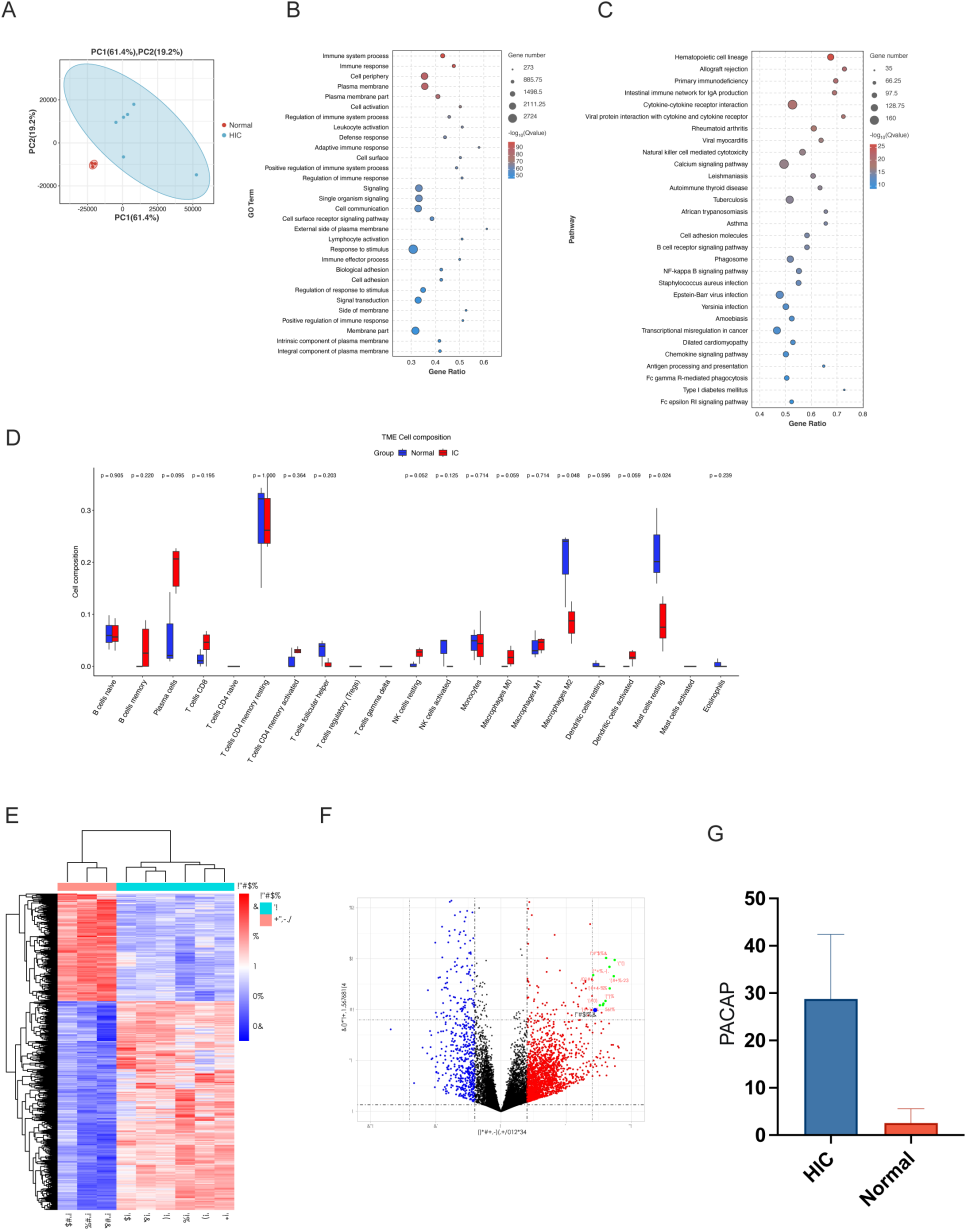
Molecular and immune analysis in IC/BPS patient samples. **(A)** PLS-DA Plot of Molecular Profiles in IC/BPS vs. Control Groups: Partial least squares-discriminant analysis (PLS-DA) plot showcasing the clear separation between normal controls and HIC patient groups, reflecting significant differences in their respective molecular profiles in human subjects. **(B)** GO Term Enrichment Analysis for IC/BPS Samples: Gene Ontology (GO) term enrichment analysis results, identifying key biological processes, molecular functions, and cellular components, predominantly associated with immune response and signaling. **(C)** KEGG Pathway Enrichment Analysis in IC/BPS Pathology: Kyoto Encyclopedia of Genes and Genomes (KEGG) pathway enrichment analysis highlighting pathways relevant to hematopoietic cell lineage and allograft rejection, suggesting an immune response involvement in interstitial cystitis/bladder pain syndrome (IC/BPS) pathology. **(D)** Immune Infiltration Analysis in IC/BPS vs. Control Groups: Immune Infiltration Analysis indicating minimal alterations in most immune cell types between IC/BPS and control groups, except for resting mast cells and monocytes, suggesting their potential role in IC/BPS pathogenesis. **(E)** Heatmap of Differential Gene Expression in IC/BPS: Heatmap representation of transcriptomic data contrasting gene expression profiles between IC specimens and normal controls, emphasizing the differential expression of ADCYAP1. **(F)** Volcano Plot of Gene Expression in IC/BPS Samples: Volcano plot illustrating the differential gene expression between control and Hunner type interstitial cystitis (HIC) patient groups. Red points represent genes that are significantly upregulated, highlighting ADCYAP1 due to its substantial upregulation in IC samples compared to controls, marking it as a prominent biomarker and potential therapeutic target for IC/BPS. Blue points indicate significantly downregulated genes, and black points denote genes without significant changes. **(G)** Bar Graph of PACAP Expression in IC/BPS vs. Control Tissues: Bar graph depicting the increased expression of PACAP in surgical specimens from IC/BPS patients compared to normal tissues, confirming its role as a diagnostic and therapeutic marker.

PLS-DA revealed discernible differences between the two sample cohorts, with minimal overlap, indicating statistically significant distinctions (**Figure 2A**).

GO analysis highlighted significant biological processes, molecular functions, and cellular components, especially those related to immune processes and cell signaling. More than 11,000 entries were associated with immune system processes. KEGG analysis demonstrated the importance of various pathways, such as Hematopoietic cell lineage and Allograft rejection, highlighting the immunological aspect of IC/BPS pathology (**Figures 2B, C**).

GSEA identified 326 enriched KEGG pathways, of which 126 were significant. Pathways related to infection and immunity, such as Autoimmune thyroid disease and Allograft rejection, were upregulated in IC, while pathways like Valine degradation were downregulated, suggesting links to IC/BPS pathology (**Supplementary Figure S1**). Overall, these analyses reveal a strong immunological component in the pathophysiology of IC/BPS, with significant upregulation and downregulation of specific pathways, offering insights into potential pathological mechanisms.

#### Immune infiltration analysis

Analysis of immune infiltration patterns within the IC/BPS group shows that, except for resting mast cells and monocytes, most immune cell types show minimal changes compared to the control group. This observation highlights the potential pivotal role of resting mast cells and monocytes in IC/BPS pathogenesis, warranting further investigation (**Figure 2D**).

In transcriptomic data analysis, a notable bifurcation in gene expression was observed using a heat map comparing six IC specimens and a cohort of three normal specimens (**Figure 2E**). The gene of interest, ADCYAP1 (commonly known as PACAP), stood out with significant distinction, showing a marked increase in expression in the IC group. This was prominently indicated by its unique presence in the upper red quadrant of the volcano plot (**Figure 2F**). This significant upregulation suggests that ADCYAP1 plays a pivotal role in IC/BPS pathogenesis, marking it as a potential biomarker and therapeutic target. The pronounced disparity in ADCYAP1 expression between IC samples and normal specimens presents a promising avenue for elucidating the complex molecular mechanisms underlying IC/BPS.

### Surgical specimen validation

Building on the insights from the transcriptomic analysis, we further validated the expression profile of PACAP (ADCYAP1) through an empirical evaluation of surgical specimens from IC/BPS patients. A collection of nine samples, consisting of three normal specimens and six IC/BPS specimens, was subjected to PACAP immunohistochemical staining assays (**Supplementary Figure S2**). The immunohistochemical data revealed a significant increase in PACAP expression within IC/BPS tissues compared to normal tissues (**Figure 2G**). This significant elevation in PACAP levels supports its potential as a diagnostic marker and therapeutic target in IC/BPS pathophysiology.

### Enhancement of bladder function following intravesical instillation of PACAP6-38/Saline

**Figure 3:**
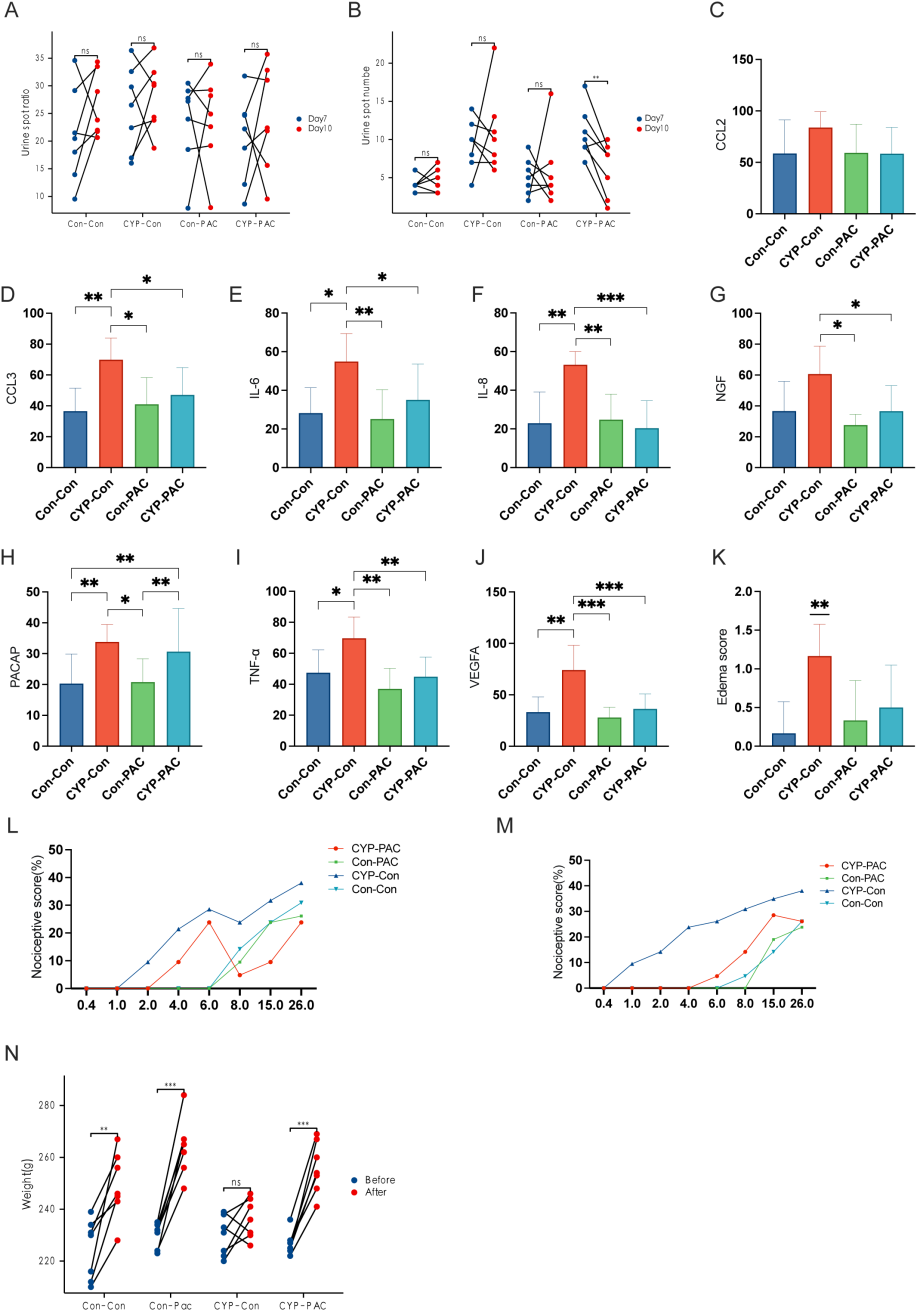
Effects of CYP and PACAP treatments on bladder inflammation and pain sensitivity in rat models. **(A, B)** Sequential Urine Spot Frequency and Area Analysis Post-Treatment: Sequential urine spot assay results indicating a significant increase in urine spot frequency in the CYP-PAC group post-treatment (P<0.05), without notable changes in urine spot area. **(C–J)** Immunohistochemical Analysis of Cytokines in Bladder Tissues: Immunohistochemical analysis of bladder tissues showing elevated levels of cytokines and growth factors—CCL3, IL-6, IL-8, TNF-α, and VEGF—in the CYP-Control group, with a significant increase in NGF levels compared to CYP-PAC and Control-PAC groups (P < 0.05). PACAP expression was similarly upregulated in the CYP-Control and CYP-PAC groups relative to the Con-PAC and Con-Con groups. **(K)** Pre-Treatment von Frey Test Response Curve: Assessment of bladder tissue edema scores, illustrating significant inflammation in the CYP-Con group compared to the Con-Con group (p < 0.01) and no significant difference between the CYP-PAC and Con-Con groups. **(L)** Post-Treatment von Frey Test Results: von Frey test response curves pre-treatment demonstrating increased sensitivity to stimuli in the CYP-treated groups. **(M)** Weight Progression of Rats Over the Experiment Period: Post-treatment von Frey test results showing a significant reduction in visceral pain in the CYP-PAC group compared to the CYP-Con group (p < 0.05). **(N)** Graph showing the weight progression of rats over the course of the experiment. Weight measurements were taken on Day 0 and Day 11 to track growth and development during the treatment period. *: p < 0.05; **; p < 0.01; ***; p < 0.001.

Building on the foundational transcriptomic sequencing analysis and subsequent immunohistochemical validation with surgical specimens, we expanded the research to animal models. We employed the Urine Spot Assay to analyze micturition patterns in SD rats. The resulting data, shown graphically, indicated that rats in the CYP-PAC cohort exhibited a statistically significant increase in urine spot frequency post-infusion compared to the pre-infusion baseline (P < 0.05). In contrast, as shown in **Table 1**, no statistical difference was observed in urine spot area before and after PACAP6-38 administration across the cohorts under investigation. Notably, the urine spot frequency in the CYP-Control group was significantly higher compared to the other three groups (**Figures 3A, B**).

**Table 1:** Comparison of urodynamic parameters and body weight ratios across control and cystitis-induced rat groups post-treatment.

characteristics	Con-Con	CYP-Con	Con-PAC	CYP-PAC	CYP-Treated	CYP-Untreated
(mL)	1.28 ± 0.38	0.63 ± 0.22***	1.272± 0.39	1.13± 0.77	1.86 ± 0.82^^	0.88 ± 0.13
Pdet(cmH2O)	41.77 ± 12.02	35.58 ± 3.04	39.02± 5.70	38.82 ± 12.15	40.52 (34.45, 43.88)	37.66 (31.92, 40.92)
BC(mL/cmH2O)	0.045 ± 0.010	0.026 ± 0.010*	0.046 ± 0.015	0.047 ± 0.037	0.058 ± 0.023^	0.033± 0.008
Baseline (cmH20)	10.96 (9.58, 12.10)	10.88 (10.34, 12.33)	9.9 (9.59, 10.26)	9.56 (9.07, 12.19)	9.81 (9.72, 9.97)	10.21 (9.47, 10.82)
Pre-Body weight(g)	224.57 ± 11.63	229.57 ± 7.68	230.14 ± 4.74	227 ± 4.47	225.14 ± 9.19	234 ± 11.55
Post-Body weight(g)	249.29 ± 12.88	236.29 ± 7.63*	264.14 ± 11.13	256 ± 10.07	263 (250.5, 267.5)^^	242 (233, 246)
Bladder weight (g)	0.12 ± 0.016	0.16 ± 0.019**	0.11 ± 0.0216	0.12 ± 0.012	0.10± 0.019^	0.127± 0.019
Bladder(g)/Body Weight(g)	0.49 (0.43, 0.52)	0.65 (0.58, 0.72)**	0.4 (0.4, 0.5)	0.5 (0.45, 0.5)	0.402 ± 0.059^^^	0.582 ± 0.088

MBC, maximal bladder capacity, Bladder Compliance (BC), Maximum Bladder Pressure (Pdet), *p values < 0.05, **p values < 0.01, ***p values<0.001 when compared with controls;^ p values between CYP-Treated and CYP-Untreated.

Further exploration involved the use of urodynamic testing to evaluate bladder function in the SD rat cohorts. The tabulated data showed that the CYP-Control group had a significant reduction in both Maximum Bladder Capacity (MBC) and Bladder Compliance (BC) compared to the other three groups (P < 0.05). However, when evaluating Maximum Bladder Pressure (Pdet) and baseline, no statistically significant differences were found in the intergroup comparisons (**Table 1**).

### Mitigation of bladder inflammation with PACAP6-38/saline intravesical instillation

The immunohistochemical findings, presented in **Figures 3C–J**, reveal a clear upregulation in the expression of several cytokines and growth factors—specifically CCL3, IL-6, IL-8, TNF-α, and VEGF—within the CYP-Control cohort, with statistically significant differences (P < 0.05). Furthermore, the concentration of Nerve Growth Factor (NGF) in the CYP-Control group was higher than levels observed in both the CYP-PAC and Control-PAC groups. Similarly, PACAP expression levels were higher in the CYP-Control and CYP-PAC groups compared to the Con-PAC and Con-Con groups. Supporting the immunohistochemical results, the edema score in the CYP-Con group was significantly higher, reinforcing our histological findings with strong statistical significance (P < 0.01). No statistical difference was observed between the CYP-PAC group and the Con-Con group (**Figure 3K**).

### Improvement of general condition following PACAP6-38/saline intravesical instillation

Nociceptive responses were assessed using the von Frey test. **Figures 3L, M** show the von Frey test performed on day 7 before treatment after the induction of chronic cystitis. At this stage, the CYP-PAC and CYP-Con groups, which received chronic CYP injections, exhibited visceral pain marked by increased responses to normally innocuous 1-8g von Frey forces (abnormal pain) and heightened reactions to noxious 10-26g von Frey forces (hyperalgesia) (**Figure 3L**). After treatment, the CYP-PAC group showed a significant reduction in chronic visceral pain induced by CYP compared to the control group (P < 0.05) and the sham group (**Figure 3M**).

As shown in **Figure 3N**, all groups except the CYP-Con group exhibited an increase in body weight at the second measurement. Additionally, the bladder weight-to-body weight ratio was significantly higher in the CYP-Con group compared to the other three groups (**Table 1**).

### Enhancement of bladder function following intrathecal instillation of PACAP6-38/saline

**Figure 4:**
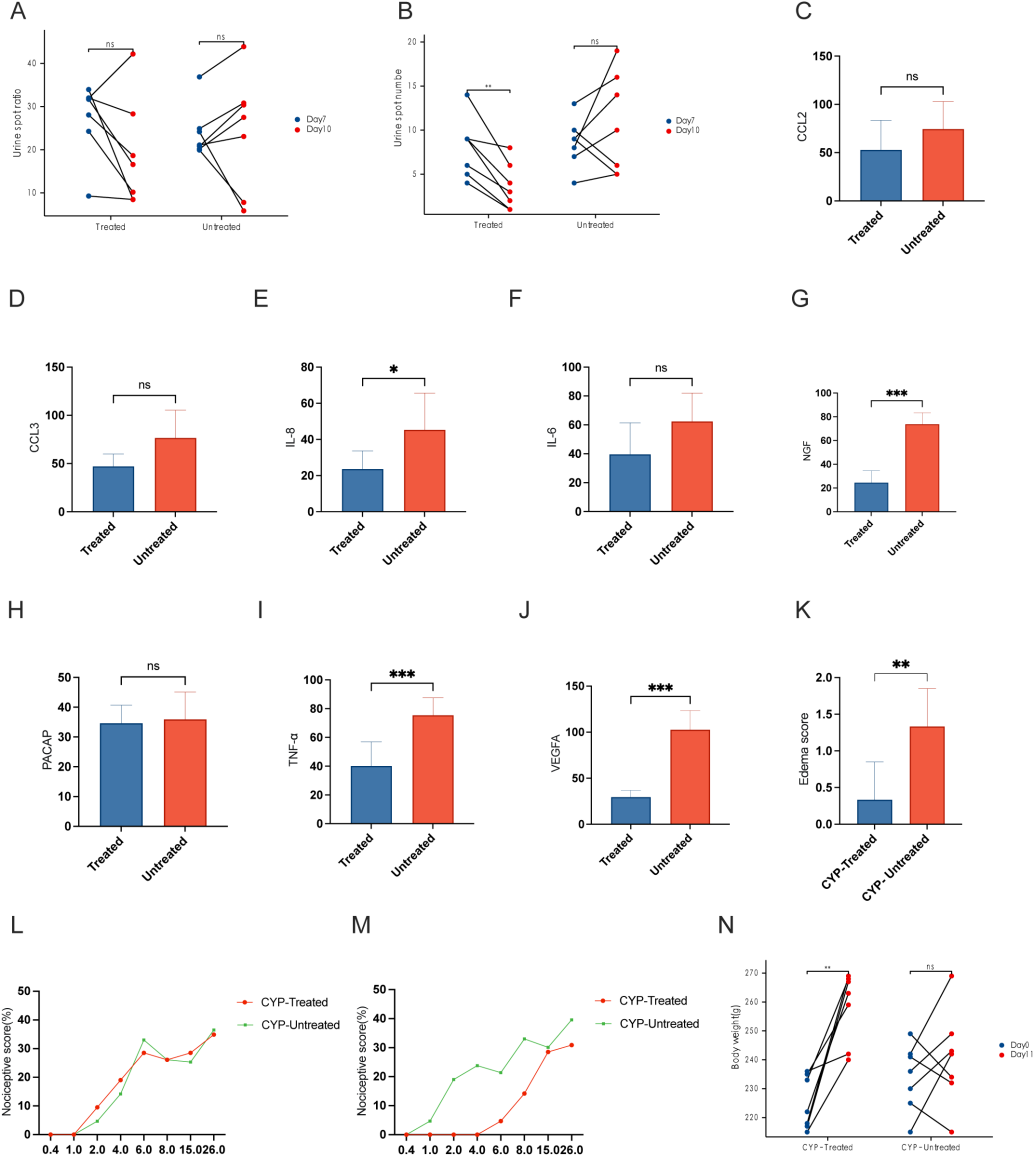
Effects of PACAP6-38 treatment on bladder function and inflammatory markers in CYP-treated rat models. **(A)** Urine Spot Size Analysis Pre- and Post-Treatment: “Comparative analysis of urine spot dimensions before and after intrathecal instillation in the CYP-treated and untreated groups. The analysis shows no significant size variation across groups, indicating that the treatment does not affect the size of urine spots. **(B)** Reduction in Urine Spot Frequency Post-PACAP6-38 Treatment: Graph depicting a reduction in urine spot frequency following intrathecal instillation of PACAP6-38 in the CYP-treated group, signifying a statistically significant improvement in bladder function (P < 0.01). This result highlights the therapeutic potential of PACAP6-38 in modulating bladder activity post-treatment. **(C–J)** Quantitative Immunohistochemical Analysis of Inflammatory Markers in CYP-Treated vs. Untreated Groups: Quantitative immunohistochemical analysis of inflammatory markers including IL-6, IL-8, NGF, TNF-α, and VEGF, indicating a significant reduction in the CYP-Treated group as opposed to the CYP-Untreated group (P < 0.05). **(K)** Bladder Tissue Edema Score: Evaluation of bladder tissue edema scores demonstrating decreased inflammation in the CYP-Treated group compared to the CYP-Untreated group (p < 0.01). **(L)** von Frey Test Response Curve Post-CYP Infusion: von Frey test response curves post-CYP infusion showing elevated sensitivity to mechanical force in both CYP-Treated and CYP-Untreated groups, with no significant difference observed between them. **(M)** Reduction in Chronic Visceral Pain Post-PACAP6-38 Treatment: A significant decrease in chronic visceral pain in the CYP-Treated group following treatment with PACAP6-38, as shown by the von Frey test (p < 0.05). **(N)** Body Weight Changes in CYP-Treated and Untreated Groups: The CYP-Treated group exhibits a significant increase in body weight after treatment (p < 0.05), in contrast to the CYP-Untreated group which showed no such difference. *: p < 0.05; **; p < 0.01; ***; p < 0.001.

**Figure 4A** shows no substantial differences in the dimensions of urine spots across the groups, both before and after the intrathecal infusion. After intrathecal administration of PACAP6-38, the CYP-treated cohort showed a significant reduction in urine spot frequency compared to their pre-injection state, with statistical significance (P < 0.01), as shown in **Figure 4B**. Furthermore, as shown in **Table 1**, the CYP-Untreated group had reduced MBC and BC compared to the CYP-Treated cohort, achieving statistical significance (P < 0.05). However, the comparison of Pdet and baseline between these cohorts did not reveal any statistically significant differences.

### Mitigation of bladder inflammation with Pacap6-38/saline intrathecal instillation

The levels of IL-6, IL-8, NGF, TNF-α, and VEGF in the CYP-Untreated group were higher than those in the CYP-Treated group, with statistically significant differences (P < 0.05) (**Figures 4C–J**). The edema score in the CYP-Untreated group was higher than that in the CYP-Treated group (P < 0.01) (**Figure 4K**).

### Improvement of general condition following PACAP6-38/saline intrathecal instillation

Following CYP infusion, both the CYP-Treated and CYP-Untreated groups showed an increase in the curves, with no statistically significant difference between the two cohorts (**Figure 4L**). After intrathecal administration of PACAP-38, the CYP-Treated group showed a significant reduction in chronic visceral pain induced by CYP post-treatment (P < 0.05) (**Figure 4M**). As shown in **Figure 4N**, the CYP-Treated group demonstrated a significant increase in weight at the second measurement (P < 0.05). In contrast, no significant difference in weight was observed before and after treatment in the CYP-Untreated group. Additionally, both bladder weight and the bladder weight-to-body weight ratio were higher in the CYP-Untreated group compared to the CYP-Treated group (**Table 1**).

## Discussion

PACAP and its receptor PAC1 play essential roles in regulating urinary tract function, particularly in conditions such as bladder pain syndrome (BPS)/interstitial cystitis (IC), which are marked by chronic pelvic pain and urinary dysfunction. PACAP is expressed in neurons in the brainstem and hypothalamus and activates preganglionic sympathetic neurons in the spinal cord during inflammatory stress. This results in PACAP influencing the immune response in the thymus, lymph nodes, and spleen through PAC1 receptors on sympathetic neurons (19). Blocking PAC1 receptors could represent a novel therapeutic strategy to improve bladder function and alleviate pelvic pain (20).

Our PLS-DA and KEGG pathway analyses revealed distinct metabolic and immunological differences in IC/BPS, supported by GO analysis that identified key immune components and highlighted specific altered pathways and genes. Notably, GSEA identified upregulation in autoimmune and infectious disease pathways, suggesting immune system dysregulation as a primary pathogenic mechanism in IC/BPS. Concurrently, downregulated metabolic pathways indicate changes in energy metabolism. Additionally, varied patterns of immune cell infiltration, particularly by mast cells and monocytes, are crucial in IC/BPS pathogenesis and warrant further investigation. Furthermore, our research establishes a strong link between PACAP expression and IC/BPS. Transcriptomic analysis revealed significant differences in PACAP expression between healthy individuals and IC/BPS patients, emphasizing its role in the disease’s immunological and potential neuroinflammatory aspects. This aligns with the broader metabolic and immunological irregularities observed in our comprehensive analyses, underscoring the complexity of IC/BPS pathophysiology.

In our study, the CYP-Con group exhibited significant bladder inflammation, indicated by increased levels of CCL3, IL-6, IL-8, TNF-α, and VEGF, highlighting the PACAP/PAC1 pathway’s role in this inflammation. This suggests that CYP treatment alone triggers significant inflammation. In contrast, the CYP-PAC group, treated with PACAP6-38, showed reduced inflammatory markers, indicating that PACAP6-38 may counter CYP-induced inflammation by inhibiting the PACAP/PAC1 pathway, likely through reducing inflammatory cell recruitment and mediator regulation. Additionally, intrathecal administration of PACAP6-38 highlighted its significant effect in the dorsal root ganglion (DRG), which is crucial for pain and inflammation management. This suggests its ability to modify pain perception and inflammatory responses, as shown by reduced pain and inflammation. This underscores its role in managing neuroinflammation in interstitial cystitis. Intravesical administration of PACAP6-38 effectively reduced bladder inflammation, supporting the PACAP/PAC1 pathway’s role in reducing inflammatory markers and improving bladder function. These findings reveal PACAP6-38’s dual action in the DRG and bladder, suggesting it alleviates interstitial cystitis symptoms by reducing neuroinflammation and pain in the DRG while directly addressing bladder inflammation to improve function.

In the CYP-PAC group, PACAP6-38 treatment significantly reduced urine spot count without affecting the spot area, suggesting an impact on bladder voiding function. This reduction in urine spot count indicates decreased bladder hyperactivity, a key clinical indicator in interstitial cystitis. Moreover, the CYP-Con group showed lower maximum bladder capacity and compliance compared to other groups, highlighting bladder dysfunction caused by CYP treatment. Conversely, the improved bladder capacity and compliance in the CYP-PAC group underscore PACAP6-38’s effectiveness in restoring bladder function affected by inflammation. Additionally, Von Frey test results in the CYP-PAC group showed a significant decrease in chronic visceral pain post-treatment, likely due to PACAP6-38’s modulation of bladder inflammation and sensory function. This result, combined with observed physiological weight gain, reinforces PACAP6-38’s role in improving overall health under chronic inflammatory conditions.

Vizzard et al. demonstrated an upregulation of PACAP expression in the DRG segments associated with the micturition reflex in cyclophosphamide-induced chronic cystitis rats (21). Braas et al. found that intrathecal injection or bladder instillation of the PAC1 antagonist PACAP (6-38) reduced voiding frequency in animals with cystitis (9). Victory May et al. noted that during detrusor muscle contraction, PACAP promotes ATP release from the urothelium. PACAP gene knockout mice showed increased bladder mass, fewer but larger urine spots in the micturition imprint test, thicker lamina propria and detrusor smooth muscle, but no significant differences in the urothelium. Additionally, PACAP gene knockout mice showed increased bladder capacity, voiding volume, significantly longer voiding intervals, prolonged detrusor muscle contraction duration, and increased residual urine volume. PACAP (+/-) heterozygous gene knockout mice also showed bladder dysfunction, albeit to a lesser extent (22). These findings suggest that PACAP mediates changes in bladder function by modulating ATP release. Recent research by Atsuko Hayata-Takano et al. showed that PACAP-deficient mice exhibited motor and cognitive abnormalities, which were improved with a 5-HT receptor antagonist. They confirmed that PACAP induces increased internalization of 5-HT (2A) in HEK293T cells, but not of 5-HT (1A), 5-HT (2C), dopamine D2 receptors, or metabotropic glutamate receptor 2, thereby attenuating 5-HT (2A)-mediated signaling. This effect was inhibited by protein kinase C inhibitors, β-arrestin2 silencing (a key protein regulating endothelial nitric oxide synthase activity), the PAC1 receptor antagonist PACAP6-38, and PAC1 silencing (23).

The urinary epithelium is a specialized epithelial tissue that lines most urinary tract structures, forming a barrier against urine components passing into underlying tissues and the bloodstream (24, 25). Damage to the urinary epithelium increases its permeability, allowing urea, potassium, and other urinary solutes to penetrate the bladder wall. This activates an inflammatory response and mast cell secretion, which subsequently increases the production of pro-inflammatory mediators such as tumor necrosis factor-alpha (TNF-α), interleukin (IL)-1β, and IL-8. These mediators sensitize nerve endings, leading to increased release of neuropeptides that promote mast cell degranulation and exacerbate the inflammatory process. Inflammation directly impacts bladder function. In acute inflammation, such as urinary tract infections (UTIs), inflammatory mediators are released, damaging the urinary epithelium and causing bladder wall irritation. These inflammatory changes result in clinical symptoms such as urgency, dysuria, frequency, nocturia, and fever. In acute inflammation, these changes are transient and resolve once the harmful stimuli are removed. If the harmful stimuli persist, they can lead to chronic inflammation, causing recurrent damage to the bladder mucosa and other functional pathological changes such as fibrosis. These pathological changes contribute to symptoms commonly seen in interstitial cystitis (IC), such as urgency, frequency, dysuria, and cystoscopic findings (26). Vascular endothelial growth factor (VEGF) is overexpressed in 58% of IC bladders (27), along with IL-6 and IL-8. The release of vasoactive and inflammatory mediators by mast cells accounts for many IC symptoms. IC is characterized by the infiltration of mononuclear cells, including macrophages, lymphocytes, eosinophils, mast cells, and plasma cells, leading to irreversible tissue damage, functional dysregulation such as fibrosis and poor compliance, detrusor muscle overactivity, and visceral hypersensitivity, resulting in chronic pain and lower urinary tract symptoms. An increase in mast cell numbers in the submucosal and detrusor layers is particularly evident in classic IC with Hunner’s ulcers (28).

Typically, the half-life of inflammatory mediators is short, but in IC, prolonged exposure to harmful stimuli leads to increased secretion of inflammatory mediators, resulting in vascular edema, vasculitis, and neuroinflammation. This process promotes neurotransmitter secretion, further stimulating mast cells and creating a vicious cycle of sustained inflammation and repeated damage to the urinary epithelium. Clinically, this manifests as visceral hypersensitivity, leading to difficulty in urination, urgency, and lower urinary tract symptoms. We speculate that pathological changes, such as the loss of the urinary epithelial glycosaminoglycan (GAG) layer, impaired immune function, and infections, lead to alterations in the PACAP/PAC1-related regulatory molecular network in the bladder, resulting in inflammatory cascades and excessive mast cell expression. This can trigger either Hunner’s ulcerative IC or non-ulcerative IC. Simultaneously, direct stimulation of the urinary epithelium by urine leads to C-fiber overactivation, resulting in chronic pain symptoms. The PACAP/PAC1 pathway further increases inflammatory mediator secretion, promotes neurotransmitter release, and stimulates mast cells, leading to sustained inflammation, repeated damage to the urinary epithelium, and the development of inflammatory cascades and bladder dysfunction.

## Conclusion

In conclusion, PACAP and its receptor PAC1 play a complex and multifaceted role in the pathogenesis of IC/BPS, influencing various physiological systems to maintain homeostasis. Our findings, along with previous research, highlight PACAP’s significance in IC/BPS, particularly regarding immune and neuroinflammatory aspects. The differential expression of PACAP observed in normal and IC/BPS patients underscores its crucial role. Modulating the PACAP/PAC1 pathway through interventions such as PAC1 antagonists has shown promise in reducing bladder inflammation and improving function. PACAP’s role in sensory nerve regulation and neurotransmitter release, along with its interaction with mast cells and inflammatory mediators, further emphasizes its importance in IC/BPS. Chronic and neurogenic inflammation associated with IC/BPS symptoms such as bladder pain and urgency are linked to dysregulated pathways influenced by factors such as immune dysfunction and infections. Our research highlights PACAP’s complex role in IC/BPS and its therapeutic potential, emphasizing the need for further investigation into its specific mechanisms and interactions to develop targeted treatments.

## Data Availability

The data presented in the study are deposited in the NCBI Sequence Read Archive (SRA) under the accession numbers SRR31282777-SRR31282785, available at https://www.ncbi.nlm.nih.gov/sra/PRJNA1183631.

## Supplementary material

Supplementary Figure 1 Gene Set Enrichment Analysis (GSEA) of KEGG pathways associated with interstitial cystitis/bladder pain syndrome (IC/BPS).

Supplementary Figure 2 Immunohistochemical Staining of Bladder Tissue for PACAP in HIC Patients and Normal Group. **(A-F)** Display PACAP immunohistochemical staining in bladder tissues from patients with Hunner type interstitial cystitis (HIC). **(G-I)**: Show PACAP staining in bladder tissues from the normal control group, serving as a baseline comparison to highlight the differential expression observed in the HIC group.
