# Supplementary figures and images for: PACAP/PAC1 regulation in cystitis rats: induction of bladder inflammation cascade leading to bladder dysfunction
Source: Front Immunol. 2024 Nov 28;15:1413078. doi: 10.3389/fimmu.2024.1413078 (PMC11634801; doi:10.3389/fimmu.2024.1413078)

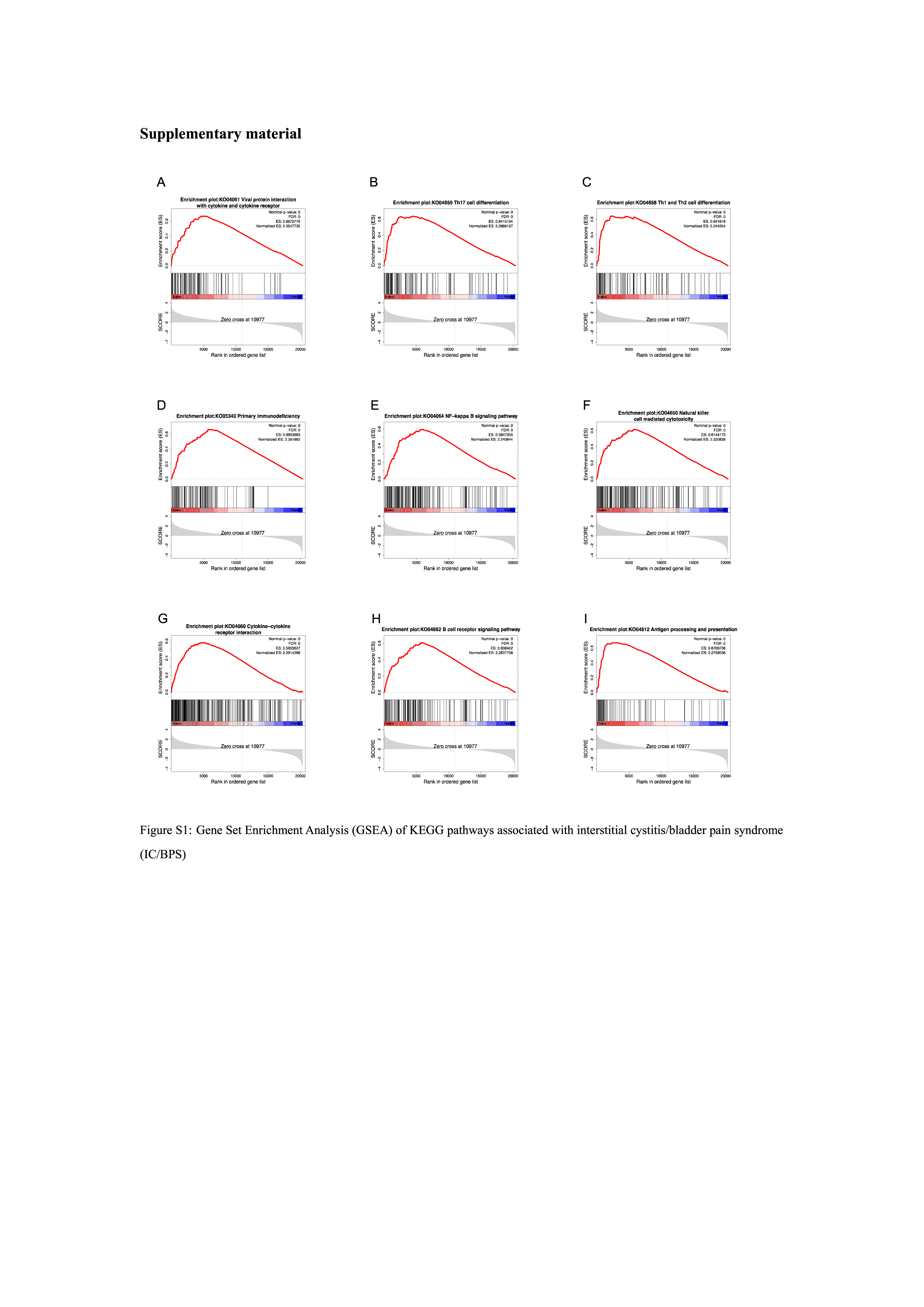

Supplement: Supplementary Figure 1 — Gene Set Enrichment Analysis (GSEA) of KEGG pathways associated with interstitial cystitis/bladder pain syndrome (IC/BPS). [file Image1.tif]

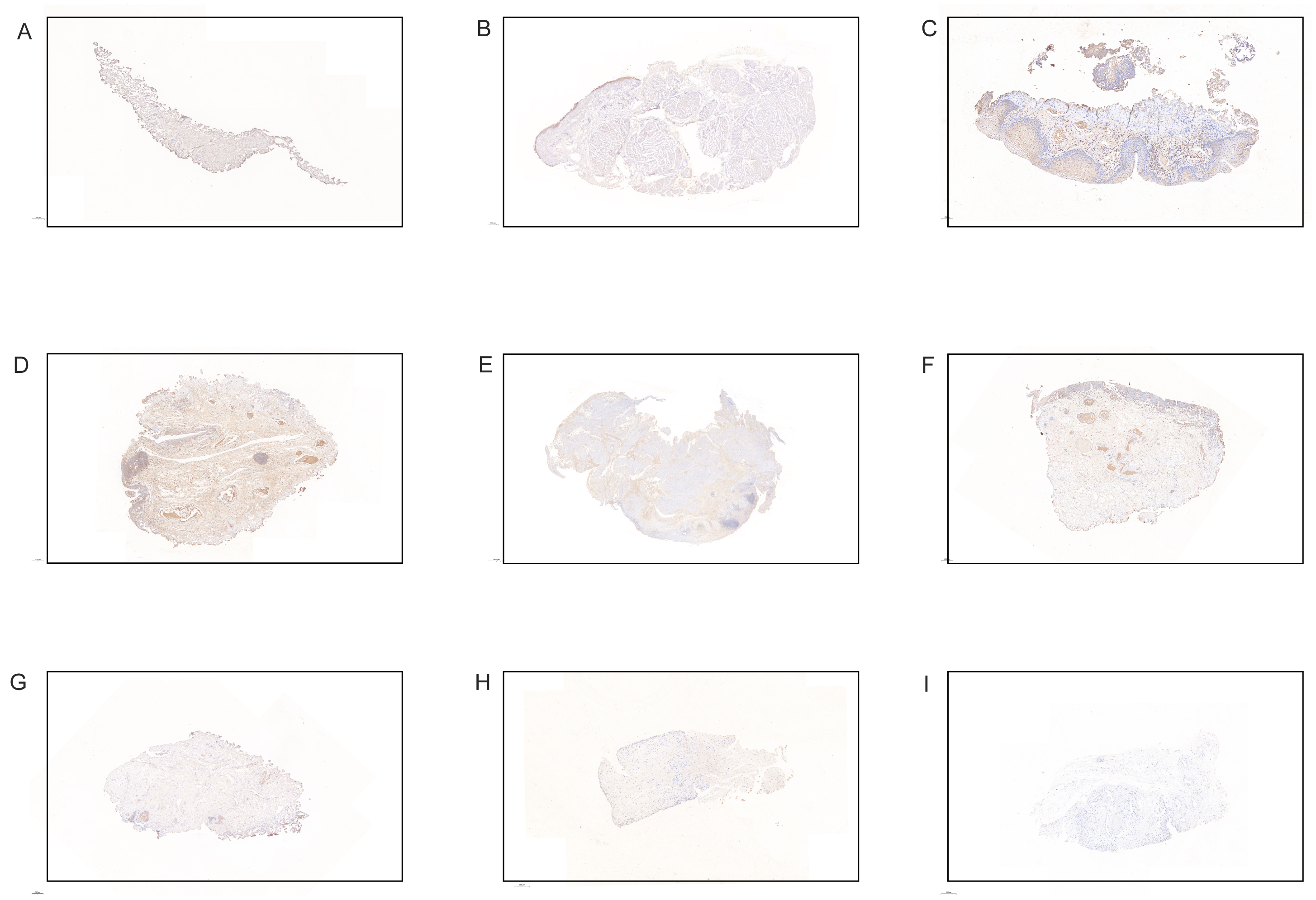

Supplement: Supplementary Figure 2 — Immunohistochemical Staining of Bladder Tissue for PACAP in HIC Patients and Normal Group. (A-F) Display PACAP immunohistochemical staining in bladder tissues from patients with Hunner type interstitial cystitis (HIC). (G-I): Show PACAP staining in bladder tissues from the normal control group, serving as a baseline comparison to highlight the differential expression observed in the HIC group. [file Image2.tif]
